# Synergistic Antibacterial Mechanism of Mannosylerythritol Lipid-A and Lactic Acid on *Listeria monocytogenes* Based on Transcriptomic Analysis

**DOI:** 10.3390/foods11172660

**Published:** 2022-09-01

**Authors:** Xiayu Liu, Xinxin Pang, Yansha Wu, Yajing Wu, Ying Shi, Xinglin Zhang, Qihe Chen

**Affiliations:** 1 Department of Food Science and Nutrition, Zhejiang University, Yuhangtang Rd. 866, Hangzhou 310058, China; 2 College of Agriculture and Forestry, Linyi University, Linyi 276005, China

**Keywords:** Mannosylerythritol lipids-A (MEL-A), lactic acid, *Listeria monocytogenes*, RNA-Seq

## Abstract

Mannosylerythritol lipids-A (MEL-A) is a novel biosurfactant with multiple biological effects. The synergistic antibacterial activity and mechanism of MEL-A and lactic acid (LA) against *Listeria monocytogenes* were investigated. The synergistic effect resulted in a significant increase in the antibacterial rate compared to LA treatment alone. Genome-wide transcriptomic analysis was applied to deeply investigate the synergistic antibacterial mechanism. Gene Ontology (GO) enrichment analysis showed that the synergy between MEL-A and LA affected many potential cellular responses, including the sugar phosphotransferase system, carbohydrate transport, and ribosomes. KEGG enrichment analysis showed that the PTS system and ribosome-related pathways were significantly enriched. In addition, synergistic treatment affected locomotion and membrane-related cellular responses in GO enrichment analysis and carbohydrate metabolism and amino acid metabolism pathways in KEGG enrichment analysis compared to LA treatment alone. The accuracy of the transcriptome analysis results was verified by qPCR (R^2^ = 0.9903). This study will provide new insights for the prevention and control of *L. monocytogenes*.

## 1. Introduction

Foodborne diseases caused by foodborne pathogens are one of the important factors affecting food safety and human health [1]. *L. monocytogenes* is a representative Gram-positive foodborne pathogen that can cause severe meningitis, sepsis, and pregnancy loss primarily in immunocompromised individuals, with a fatality rate as high as 20–30% [2]. Most fatal cases of listeriosis are primarily through the ingestion of contaminated processed food products, raw meat, sea food, dairy products, etc. [3]. *L. monocytogenes* can also utilize a plethora of complex regulatory mechanisms (e.g., riboregulators and small non-coding RNAs) to rapidly adapt to different extreme environments and can traverse different epithelial barriers to cause host infection [2,4]. Hence, the exploration of biochemical agents that effectively inhibit the growth and survival of *L. monocytogenes* is crucial for food safety.

Lactic acid is a “Generally Recognized as Safe” (GRAS) food additive and has been widely used for the control of pathogenic bacteria in perishable foods such as meat and dairy products due to its simple process, significant antibacterial effect, and low price [5,6]. LA has been reported to have significant antibacterial activity against pathogens such as *Salmonella enterica*, *Escherichia coli,* and *L. monocytogenes* [7]. Previous studies have shown that LA can lead to the destruction of the membrane structure and intracellular structure of pathogenic bacteria and the leakage of proteins, affecting the physiological and morphological changes of bacterial cells to play an antibacterial effect [7,8]. However, due to the complexity of the composition of real food systems and the strong tolerance of *L. monocytogenes*, a high addition level is often required to effectively inhibit *L. monocytogenes* in food systems [9]. Moreover, the addition of LA at high levels may affect the color, sensorial properties, and commercial characteristics of food products [10]. Therefore, *L. monocytogenes* can be effectively controlled in real food systems through the synergistic use of antimicrobial agents, while reducing the concentration of individual use [11].

Mannosylerythritol lipids (MELs) are glycolipid biosurfactants mainly produced by yeast strains of the genus *Pseudozyma* [12]. Depending on the degree of acetylation, MELs can be classified into four different conformations, including MEL-A, MEL-B, MEL-C, and MEL-D [13]. MELs exhibit not only excellent interfacial activity and biodegradability, but also biochemical activities such as antioxidant, antibacterial, anti-inflammatory, and anticancer [14]. In terms of antibacterial activity, our previous studies showed that MELs exhibited excellent inhibitory effects on Gram-positive pathogens such as *Staphylococcus aureus* and *Bacillus cereus* [15,16,17]. In terms of the antibacterial mechanism, our previous studies have shown that MEL-A has a significant damaging effect on the cell membrane of pathogenic bacteria, causing the leakage of intracellular nucleic acids and proteins and affecting the expression of the ABC transport system and membrane-related genes [16,18]. In addition, studies have shown that MEL-A can significantly improve the antibacterial activity against methicillin-resistant *Staphylococcus aureus* (MRSA) in the biofilm state and *L. monocytogenes* in the planktonic state by synergistic treatment with physical means, such as ultrasound and ultrahigh pressure [19,20].

There are also differences in the gene expression and regulation patterns of bacteria under different stresses. For instance, a pulsed magnetic field (PMF) can lead to the death of *L. monocytogenes* primarily through disruption of biological functions (phosphorylation and dephosphorylation, membrane, quorum sensing, etc.) and metabolism (carbohydrate metabolism, energy metabolism, amino acid metabolism, etc.) [21]. Fingered Citron essential oil exerts anti-listeria activity mainly by affecting chemotaxis and flagellar assembly as well as carbohydrate and metal cation uptake [22]. Furthermore, the regulation of genes under the same stress may have strain differences [23]. However, the synergistic antibacterial activity of MEL-A with chemical agents has been little explored. Therefore, in this study, we focused on the synergistic antibacterial activity of MEL-A and LA against *L. monocytogenes* EGD-e, a representative 1/2a serotype associated with human infection [24] and explored the antibacterial mechanism of synergistic processing through genome-wide transcriptomic analysis.

## 2. Materials and Methods

### 2.1. Bacterial Strains and Chemical Reagents

*L. monocytogenes* EGD-e (ATCC BAA-679, serovar 1/2a) was purchased from the American Type Culture Collection (ATCC, Manassas, VA, USA). Unless otherwise mentioned, *L. monocytogenes* was cultured in brain heart infusion (BHI) or BHI agar (Hopebiol, Qingdao, China) at 37 °C. Lactic acid (purity, over 98%, CAS 79-33-4) was purchased from Aladdin (Shanghai, China) and dissolved in sterile water. MEL-A was produced by our laboratory according to the previous method [18,19,25].

### 2.2. Synergistic Antibacterial Activity Assessment

To evaluate the synergistic antibacterial activity of MEL-A and LA against *L. monocytogenes*, 50 mL of BHI with different concentrations of LA (0, 1, and 2 mg/mL) and MEL-A (0 and 32 μg/mL) was prepared. Then, logarithmic growth stage *L. monocytogenes* was harvested, washed 3 times (4000× *g*, 2 min, 37 °C) with phosphate-buffered saline (PBS, 20 mM, pH 7.4), and diluted to obtain a final inoculum of 10^6^ CFU/mL as seeds. Next, 1 mL of *L. monocytogenes* (1.0 × 10^6^ CFU/mL) seed solution was inoculated into shaking flasks (50 mL BHI), and all flasks were incubated in an orbital shaker (180 rpm at 37 °C) (Thermo Fisher Scientific, Waltham, MA, USA). Then, 100 μL of bacterial suspension was removed from the flasks after 24 h, respectively, and the gradient dilution was spread evenly on BHI plates and incubated in an incubator (Thermo Fisher Scientific, Waltham, MA, USA) at 37 °C. *L. monocytogenes* was counted by the plate count method, and the amount of bacteria was expressed as CFU/mL. The antibacterial rate was calculated according to the following formula:

R (%) = (C − E)/C × 100%(1)

where R: antibacterial rate (%); C: number of bacteria in the control group (CFU/mL); and E: the number of bacteria in the experimental group (CFU/mL).

### 2.3. RNA Isolation and Sequencing

Approximately 1 × 10^6^ CFU of *L. monocytogenes* was incubated in BHI and BHI supplemented with MEL-A (32 μg/mL) and LA (2 mg/mL) at 37 °C for 12 h. Bacterial cells were then collected by centrifugation at 2500× *g* for 5 min and then snap-frozen in liquid nitrogen. Next, total RNA was extracted using the method described by Kafantaris [26]. Then, total RNA from all samples was sent to Novogene company (Beijing, China) for subsequent library construction and transcription sequencing. Briefly, the libraries were constructed using the NEBNext^®^ UltraTM RNA Library Prep Kit (New England Biolabs, Los Angeles, CA, USA) according to the manufacturer’s instructions. Then, these libraries were sequenced on the Illumina Novaseq platform (Illumina, San Diego, CA, USA) and 150 bp paired-end readings were generated. Three biological replicates were performed in both the experimental group and the control group. Next, in-house perl scripts were used to process raw data (raw reads) in fastq format. The next step was to remove readings containing an adapter, readings containing N bases, and readings of low quality from the raw data in order to obtain clean data (clean readings). Furthermore, Q20, Q30, and GC content clean data were calculated. All downstream analyses relied on clean data.

### 2.4. Differential Expression Analysis

Differential expression analysis was performed using the DESeq R package (1.20) (Sydney, NSW, Australia). The DESeq program provides statistical routines for analyzing digital gene expression data using a negative binomial distribution model. Using Benjamini-Hochberg’s approach, we adjusted the resulting *p*-values for false discovery. Differentially expressed genes were identified by DESeq with an adjusted *p*-value of 0.05.

### 2.5. GO and KEGG Analysis of Differential Gene Expression

GO enrichment analysis of differentially expressed genes was implemented by the GOseq R package (Beijing, China). The differentially expressed genes were considered significantly enriched for GO terms with corrected *p*-values less than 0.05. KOBAS software (Beijing, China) was used to test the statistical enrichment of differentially expressed genes in KEGG pathways.

### 2.6. Real-Time qPCR Analysis

Eight genes with significant regulations were selected for RT-qPCR analysis. A PrimeScript RT reagent Kit with gDNA Eraser (Takara, Osaka, Japan) was used to synthesize cDNA. Next, real-time PCR analysis on these cDNA was performed using the TB Green^®^ Premix Ex Taq™ (Tli RNaseH Plus) Kit (Thermo Fisher Scientific, Waltham, MA, USA) and the Applied Biosystems™ QuantStudio™ 3 instrument (Thermo Fisher Scientific, Waltham, MA, USA). As an internal reference gene, *drm* was used. Data analysis was carried out using the 2^−ΔΔCt^ method [27] with 3 biological replicates per group.

### 2.7. Statistical Analysis

Data were presented as mean ± standard deviation. All experiments were analyzed using one-way analysis of variance (ANOVA) and Duncan’s multiple range test (DMRT). Tests were run in triplicate, and statistical significance was defined as *p* < 0.05.

## 3. Results and Discussion

### 3.1. Synergistic Antibacterial Activity of MEL-A and LA on L. monocytogenes

Our previous study [18] showed that MEL-A inhibited *L. monocytogenes* significantly when added at a concentration of 32 μg/mL; therefore, we chose 32 μg/mL as the addition concentration in this study. As shown in Table 1, when LA was 1 mg/mL, the addition of MEL-A could increase the antibacterial rate from 50.0% to 99.4%, and when LA was 2 mg/mL, the antibacterial rate increased from 89.4% to 99.9%; the results also showed that there was a concentration-dependent effect of LA. Previous studies have shown that the Minimal inhibitory concentrations (MIC) of LA alone against *L. monocytogenes* is 5000 μg/mL (0.5%) [28]. Therefore, the addition of MEL-A can significantly reduce the use of LA and reduce the impact on food quality. In conclusion, the synergistic treatment of MEL-A and LA significantly improved the antibacterial effect on *L. monocytogenes*.

### 3.2. Effect of Synergistic Effect of MEL-A and LA on the Transcriptome Profile of L. monocytogenes

#### 3.2.1. Global Response of *L. monocytogenes* to the Synergistic Treatment (ML Group)

**Figure 1:**
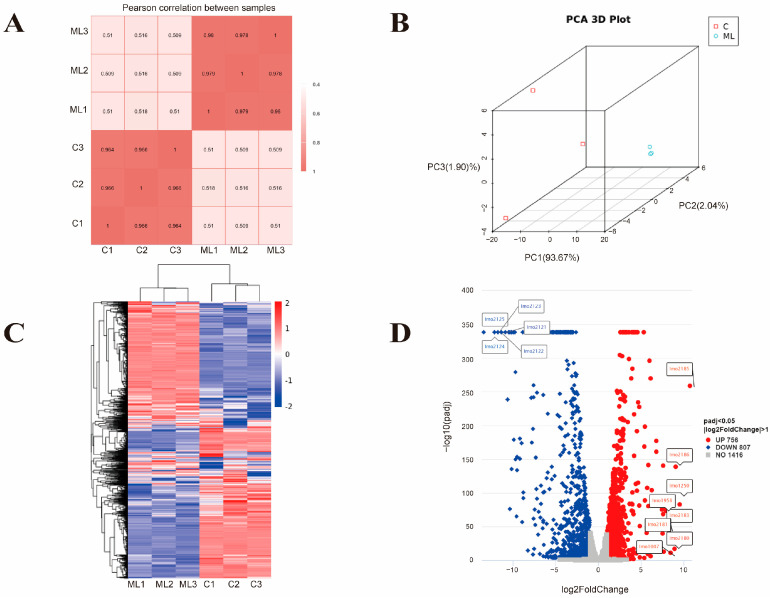
Gene expression patterns of control and ML. (**A**) Gene expression correlation graph. (**B**) Principal component analysis (PCA) graph of gene expression. (**C**) Heat map of DEGs (|log2FC| > 1 and Padj < 0.05). (**D**) Volcano plot of the DEGs (|log2FC| > 1 and Padj < 0.05). Each dot represents a gene, and the up-regulated and down-regulated genes with the largest fold change are marked in the graph. Fold change is represented on the x-axis, while p adjust is represented on the y-axis. Red and blue indicate differentially up-regulated and differentially down-regulated genes, respectively, while gray dots indicate genes that were not significantly different from the control group.

Transcriptomic analysis was performed to further study the synergistic antibacterial mechanism of MEL-A and LA against *L. monocytogenes*. As shown in Figure 1A, the squares of the Pearson correlation coefficients (R^2^) of different samples between the same group were greater than 0.96, which indicated a very high similarity of expression patterns between samples [29]. In the principal component analysis (PCA), 93.67% of the difference between the two groups came from PC1 and 2.04% of the difference came from PC2, indicating that the synergistic treatment group had a significant difference against *L. monocytogenes* compared to the control (Figure 1B). In addition, significant differences in the heat map (Figure 1C) between the two groups also indicated significant differences in expression patterns between the synergistic treatment group and the control group.

As shown in Figure 1D, a total of 1563 genes were identified as differentially expressed genes (DEGs), of which 756 were up-regulated and 807 were down-regulated (Table S1). In the synergistic treatment group, the genes *lmo2185* (log2FC 10.68), *lmo1250* (log2FC 9.49), *lmo2186* (log2FC 9.05), *lmo2180* (log2FC 8.90), *lmo1007* (log2FC 8.42), *lmo1958* (log2FC 8.42), *lmo2181* (log2FC 8.17), and *lmo2183* (log2FC 7.88) were among the top up-regulated genes. These genes mainly encode the antibiotic resistance protein, the ferrichrome ABC transporter permease, and the hypothetical protein, and they mainly belong to the Iron Transport-associated domain, Major Facilitator Superfamily (MFS), and FecCD transport family. Iron acquisition and utilization by *L. monocytogenes* is important not only for infection but also for growth and survival in diverse environments [30,31]. Furthermore, MFS transporters are also reported to be involved in the transmembrane uptake of small molecules and the excretion of harmful compounds [32]. Together, these findings suggest that the synergistic processing of MEL-A and LA may hinder the transmembrane transport of small molecules such as iron by bacteria, resulting in a decrease in bacterial viability.

The most significantly down-regulated genes mainly included an operon from *lmo2121* to *lmo2125* (log2FC ranged from −10.94 to −13.44). These genes mainly encode the maltose phosphorylase, maltodextrose utilization protein MalA, sugar ABC transporter permease, and sugar ABC transporter substrate-binding protein. These findings suggest that synergistic treatment can inhibit the sugar transport and utilization (which are reported to be critical for bacterial energy supply and growth) [33] of *L. monocytogenes*. Therefore, this may be another important reason for the synergistic effect of MEL-A and LA on *L. monocytogenes*.

#### 3.2.2. GO Enrichment Analysis of the ML Group and the Control Group

**Figure 2:**
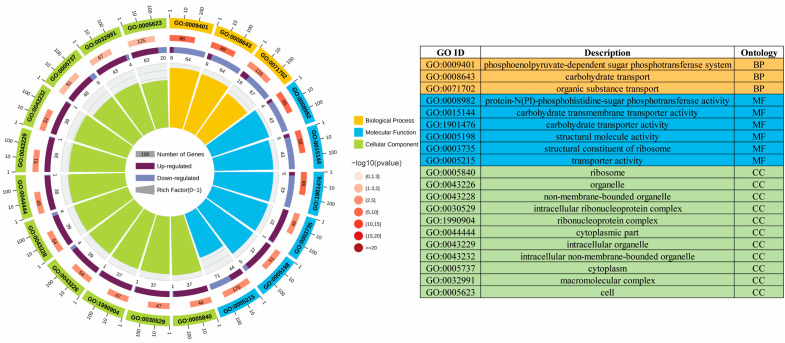
GO enrichment analysis of the ML group and the control. There are four circles from the outside to the inside. The first circle shows the classification of enrichment. Outside the circle is a coordinate ruler showing the number of genes. Each color represents a different category. On the second circle, the category number and the *p*-value are shown. A larger number of genes, a longer bar, and a smaller value results in a redder color. In the third circle, you can see the ratio of genes that are up-regulated and down-regulated; dark purple represents up-regulated genes, and light purple represents down-regulated genes. The fourth circle: the Rich Factor represents the number of foreground genes in each category divided by the number of background genes, while each cell on the background auxiliary line represents 0.1.

Our first step was to perform GO enrichment analysis in order to better understand the biological function of DEGs. The top 20 enriched GO terms of DEGs are shown in Figure 2. The most significantly enriched GO terms were “phosphoenolpyruvate-dependent sugar phosphotransferase system” in the Biological Processes (BP) category, “protein-N(PI)-phosphohistidine-sugar phosphotransferase activity” in the Molecular Function (MF) category, and “ribosome” in the Cellular Component (CC) category. The sugar phosphotransferase system has been reported to not only play a crucial role in carbohydrate uptake but also to control carbon and nitrogen metabolism, biofilm formation, and other bacterial responses to changing environmental conditions through a highly complex carbohydrate sensor system [34,35]. In addition, ribosome-related genes are also frequently identified under extreme environmental conditions (including acidity, low temperature, and high pressure, etc.) [19,36,37]. In detail, in the CC category, the most significantly enriched GO terms were “ribosome”, “intracellular ribonucleoprotein complex”, and “ribonucleoprotein complex” in the up-regulated genes (Figure S1A) and “bacterial-type flagellum” and “cell projection” in the down-regulated genes (Figure S1D). In the MF category, the most significantly enriched GO terms were “structural constituent of ribosome”, “structural molecule activity”, and “GTP binding” in the up-regulated genes (Figure S1B) and “protein-N(PI)-phosphohistidine-sugar phosphotransferase activity”, “carbohydrate transmembrane transporter activity”, and “carbohydrate transporter activity” in the down-regulated genes (Figure S1E). Moreover, in the BP category, the most significantly enriched GO terms were “cellular amide metabolic process”, “translation”, and “amide biosynthetic process” in the up-regulated genes (Figure S1C) and “phosphoenolpyruvate-dependent sugar phosphotransferase system”, “carbohydrate transport”, and “organic substance transport” in the down-regulated genes (Figure S1F).

#### 3.2.3. KEGG Pathway Enrichment Analysis of the ML Group and the Control Group

**Figure 3:**
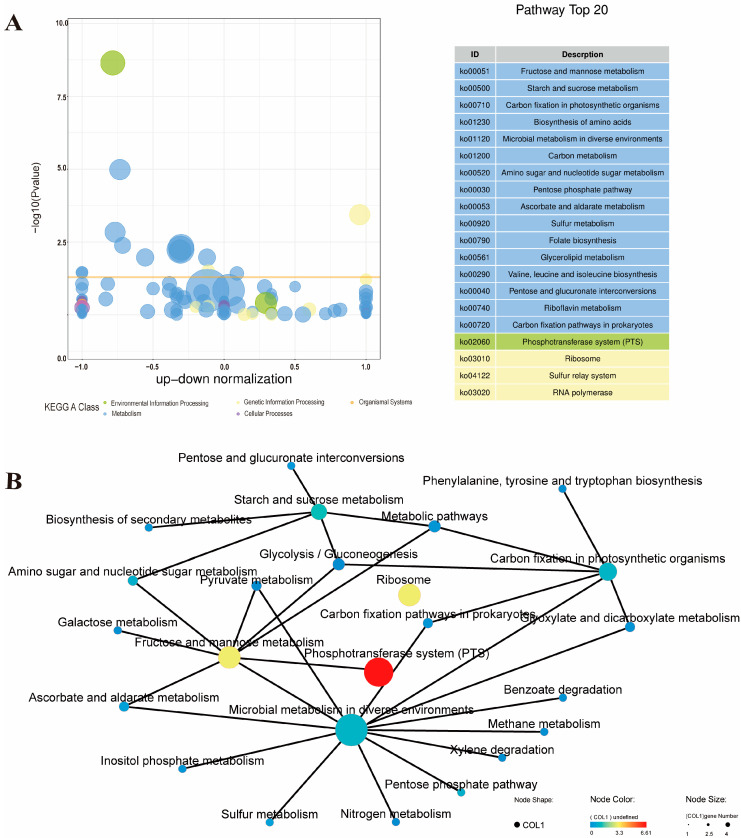
KEGG enrichment analysis of the ML group and the control. (**A**) Bubble plot of the top 20 KEGG enriched pathways. The horizontal axis of the bubble chart is the normalization coefficient of up and down; the vertical axis is −log10Pvalue. Different colors represent KEGG A classification of different pathways, and the orange threshold line is *p*-value = 0.05. Genes enriched by the current pathway are represented by the size of the bubble. The table on the right shows the 20 KEGG pathways with the smallest *p*-values. (**B**) Pathway networks enriched with KEGG annotations. The KEGG pathway is represented by nodes of different colors and sizes, with the size of the node representing the number of genes enriched in the pathway, and the gradient color of the node represents the *p*-value of the KEGG enrichment analysis; the solid line represents the connection between the pathway and the pathway or between the pathway and the gene. An isolated node in the graph indicates that the pathway is not directly related to other pathways in the graph.

KEGG pathway enrichment analysis was conducted for further in-depth analysis of DEGs. From the overall pathway, the synergistic processing of MEL-A and LA significantly affected the phosphotransferase system (PTS), ribosomes, and multiple metabolism-related pathways (including fructose and mannose metabolism, starch and sucrose metabolism, and amino sugar and nucleotide sugar metabolism, etc.) (Figure 3A). In addition, these pathways are closely related each other (Figure 3B). Specifically, among the up-regulated genes, the most significantly enriched KEGG B-level pathways were membrane transport, carbohydrate metabolism, and translation (Figure S2A), while subdivided into metabolic pathways were ribosomes, fatty acid metabolism, fatty acid biosynthesis, and ABC transporters (Figure S2B). Among the down-regulated genes, the most significantly enriched KEGG B-level pathways were membrane transport, carbohydrate metabolism, and energy metabolism (Figure S2C), while subdivided into metabolic pathways were phosphotransferase system (PTS), fructose, mannose metabolism, and starch and sucrose metabolism (Figure S2D).

**Figure 4:**
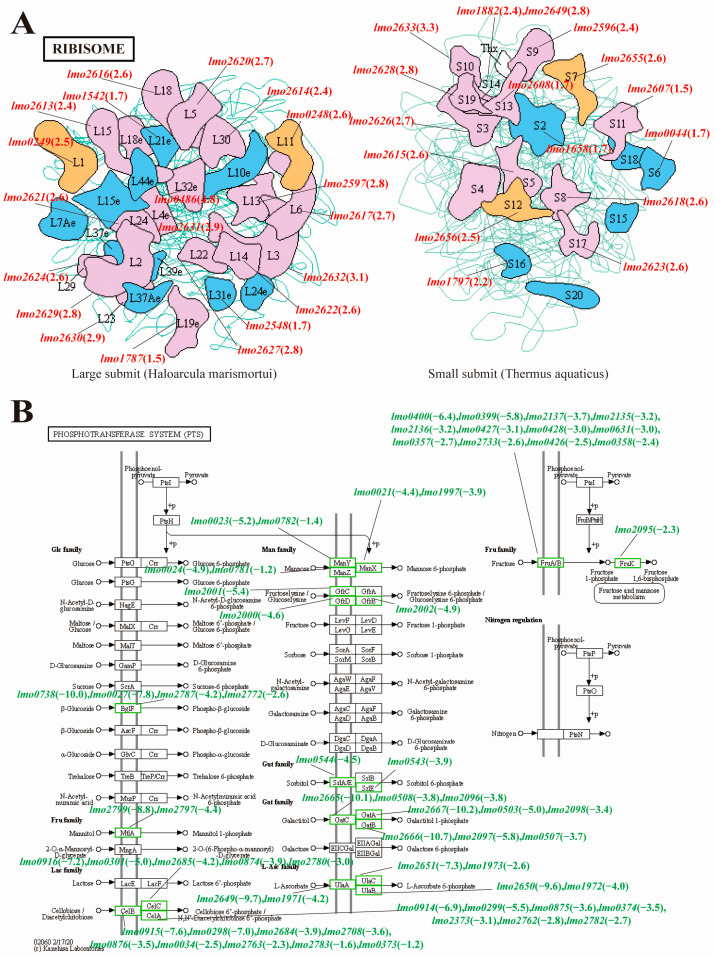
KEGG pathway analysis of ribosomes (**A**) and PTS (**B**). The red fonts in the figure represent significantly up-regulated genes in related pathways, and the green fonts represent significantly down-regulated genes in related pathways.

In this study, the ribosome was the most significantly up-regulated pathway in the KEGG enrichment analysis. The 70S ribosome of bacteria consists of a large subunit (50S) and a small subunit (30S). The large subunit contains 23S RNA, 5S RNA, and more than 30 kinds of proteins, and the small subunit contains 16S RNA and more than 20 kinds of proteins [38]. Previous studies have shown that ribosomes, as the hub of protein quality control whose main function is to convert the genetic code into amino acid sequences and build protein polymers from amino acid monomers, are essential for bacterial protein synthesis and normal life activities [39]. In addition, studies have found that many clinically useful antibiotics exert their antibacterial effects by blocking protein synthesis on bacterial ribosomes, and people have begun to artificially design some ribosome-targeting antibiotics [40,41]. Therefore, the general up-regulation of ribosome-related genes (Figure 4A) caused by the synergistic processing of MEL-A with LA suggests that the effect on the protein synthesis process of the ribosome may be an important reason for its synergistic antibacterial activity.

The phosphotransferase system (PTS) was the most significantly down-regulated pathway in the KEGG enrichment analysis. In bacteria, PTS not only catalyzes sugar transport and sugar phosphorylation, but it also regulates various transport, metabolic, and mutagenesis processes and the expression of numerous genes [42]. Many previous studies have shown that PTS is one of the key targets of many antibacterial agents [43,44,45]. As shown in Figure 4B, the synergistic treatment of MEL-A and LA also led to the downregulation of a large number of PTS-related genes, indicating that the effect of synergistic treatment on PTS sugar transport and sugar phosphorylation is also one of the important reasons for its inhibition of *L. monocytogenes*. These results are also consistent with our previous findings [19].

In this study, we selected *L. monocytogenes* EGD-e, a representative 1/2a serotype associated with human infection, for further study. However, there may be differences in the antibacterial mechanisms among strains [23], so subsequent studies could be conducted for comparative analysis of multiple strains. Moreover, previous studies have shown that both MEL-A and LA have significant damaging effects on the cell membrane structure of pathogenic bacteria and lead to the leakage of intracellular nucleic acids and proteins [7,8,16,18]. Therefore, the significant antibacterial activity of MEL-A and LA co-treatment against *L. monocytogenes* may be mainly due to the superimposed effect on the membrane structure and intracellular macromolecular substances.

### 3.3. Comparative Transcriptome Analysis of the ML Co-Treatment Group and the LA Treatment Group Alone

**Figure 5:**
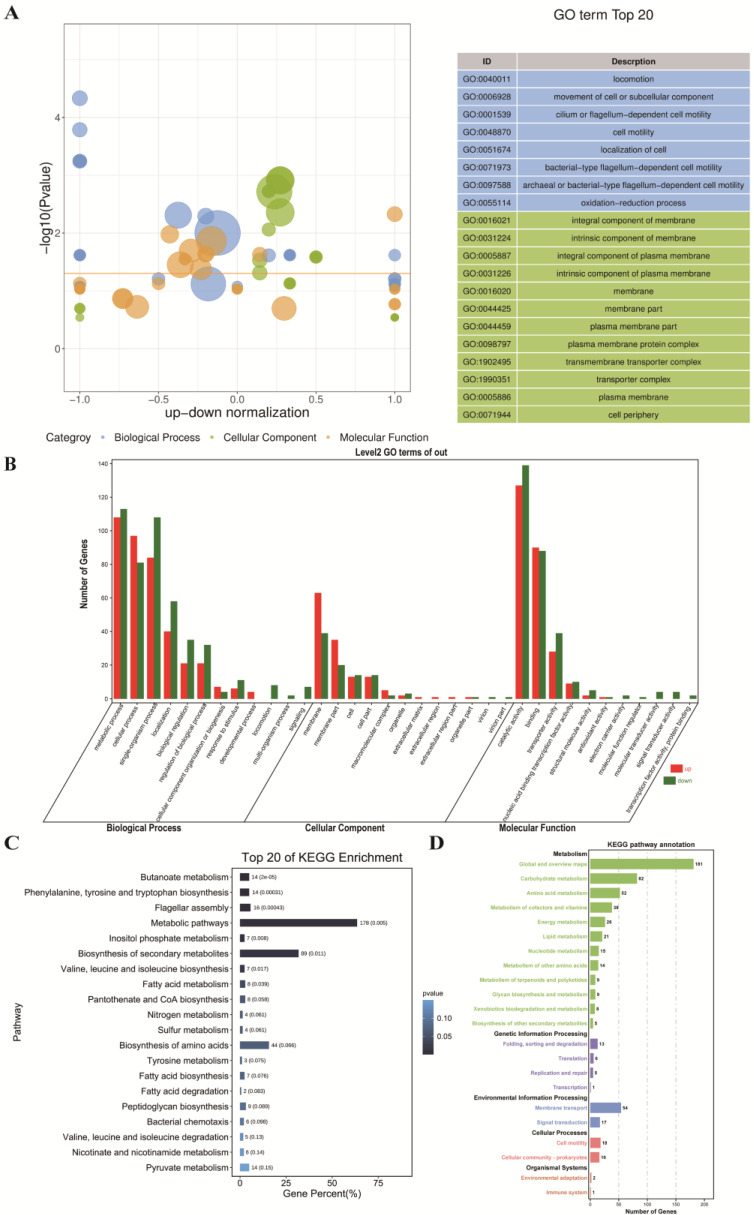
GO enrichment analysis (**A**,**B**) and KEGG enrichment analysis (**C**,**D**) of the ML co-treatment group and the LA treatment group. (**A**) Regarding the z-score bubble chart of GO enrichment analysis, the ordinate is −log10 (*p* value), and the abscissa is the up–down normalization value (the ratio of the difference between the number of differentially up-regulated genes and the number of differentially down-regulated genes to the total differential genes). The size of the bubble represents the number of target genes enriched by the current GO term; the yellow line represents the threshold of *p*-value = 0.05; the right side is the list of the top 20 terms with the *p*-value, and different colors represent different Ontologies. (**B**) Statistical plot of secondary classifications for GO enrichment analysis. The abscissa is the different GO terms, and the ordinate is the number of target genes enriched by the current GO term. Genes that are up-regulated are represented by red, while genes that are down-regulated are represented by green. (**C**) Bar graph of the top 20 significantly enriched pathways. (**D**) Statistical chart of the B-class classification for each pathway.

To further explore the addition of MEL-A on the antibacterial mechanism of *L. monocytogenes* compared with LA treatment alone, we also performed GO and KEGG enrichment analysis of the DEGs in the ML group and the LA group. In the GO enrichment analysis, locomotion-related biological processes and membrane-related (including integral components of membranes, intrinsic components of membranes, integral components of plasma membranes, intrinsic components of plasma membranes, membranes, etc.) cellular components were significantly enriched (Figure 5A). In addition, in the secondary classification of GO terms, biological processes related to metabolic processes, cellular components related to membranes, and molecular functions related to catalytic activity contained the largest number of DEGs (Figure 5B). The destruction of cell membrane structures by MEL-A has also been confirmed in our previous microstructure observation and biochemical index determination, which may be one of the key mechanisms of its antibacterial effect [14,19]. In addition, the general downregulation of locomotion-related genes indicated that MEL-A significantly reduced the motility of *L. monocytogenes*, thereby hindering its growth and reproduction process. In the KEGG enrichment analysis, pathways related to carbohydrate metabolism (butanoate metabolism, inositol phosphate metabolism, etc.) and amino acid metabolism (phenylalanine, tyrosine, and tryptophan biosynthesis, etc.) were significantly enriched. These results demonstrated that cells required carbohydrates and amino acids to repair damage caused by MEL-A treatment [21].

### 3.4. RT-qPCR Validation of RNA-Seq Data

**Figure 6:**
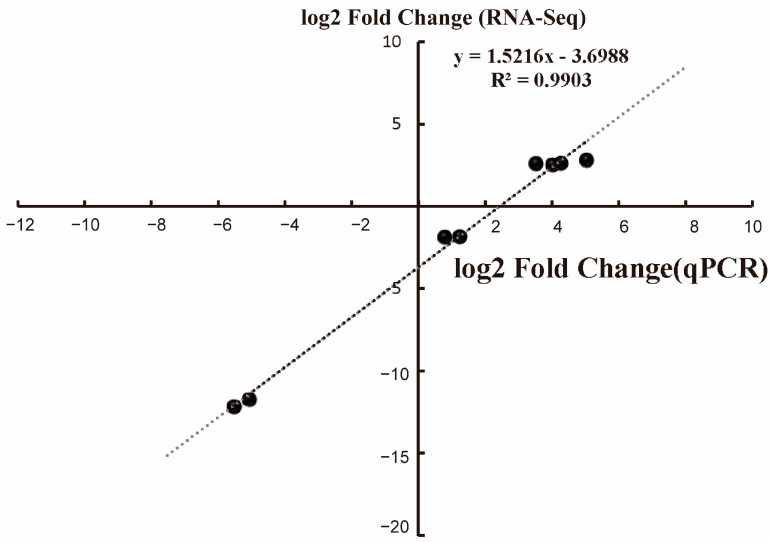
RT-qPCR validation of RNA-Seq results for the ML treatment groups. Three biological replicates were performed for each group. The horizontal coordinate is the log2 Fold Change value obtained from qPCR data and the vertical coordinate is the log2 Fold Change value obtained from RNA-Seq data.

As shown in Figure 6, we analyzed the transcription level of *lmo0248*, *lmo0249*, *lmo2623*, *lmo2628*, *lmo2123*, *lmo2124*, *lmo2347,* and *lmo2348* by real-time quantitative PCR to verify the accuracy of the transcriptome data. It was highly consistent between the RNA-Seq and qPCR results (R^2^ = 0.9903), which demonstrated the reliability of our transcriptome data.

## 4. Conclusions

In this study, the synergistic antibacterial effect of LA with a novel biosurfactant MEL-A was investigated. In addition, the gene expression changes of *L. monocytogenes* after synergistic treatment were evaluated by transcriptomics to reveal the synergistic antibacterial mechanism. Specifically, GO enrichment and KEGG pathway analysis demonstrated significant changes in sugar phosphotransferase system, carbohydrate transport, and ribosomes. Interestingly, synergistic treatment of MEL-A with LA also affected locomotion and membrane-related GO terms as well as carbohydrate metabolism and amino acid metabolism-related pathways, compared with LA treatment alone. Overall, these findings provide new insights into the synergistic antibacterial mechanisms of MEL-A and LA on *L. monocytogenes* and may contribute to the development of novel strategies against *L. monocytogenes* in the food industry.

## Figures and Tables

**Table 1:** Synergistic antibacterial activity of MEL-A and LA on *L. monocytogenes*.

Treatment	Log (CFU/mL)	Log Reduction (CFU/mL)	Antibacterial Rate (%)
Control	9.44 ± 0.01 ^a^	—	—
MEL-A (32 μg/mL)	8.70 ± 0.03 ^c^	0.74	81.8
LA (1 mg/mL)	9.13 ± 0.10 ^b^	0.31	50.0
LA (1 mg/mL) + MEL-A (32 μg/mL)	7.14 ± 0.26 ^e^	2.30	99.4
LA (2 mg/mL)	8.46 ± 0.04 ^d^	0.98	89.4
LA (2 mg/mL) + MEL-A (32 μg/mL)	6.45 ± 0.09 ^f^	2.99	99.9

Statistically significant differences (*p* < 0.05) are indicated by letters above each column (mean ± standard deviation).

## Data Availability

The raw RNA-seq data are available at NCBI Sequence Read Archive (NCBI SRA) under BioProject accession no. PRJNA862260. https://dataview.ncbi.nlm.nih.gov/object/PRJNA862260?reviewer=jgmcb3drj6q4jpdgd91u0qeq.

## Supplementary Materials

The following supporting information can be downloaded at: https://www.mdpi.com/article/10.3390/foods11172660/s1, Table S1: DEGs in the co-treated group of MEL-A and LA. Figure S1: Bubble plot of GO enrichment analysis for ML group and control. Figure S2: KEGG histogram enrichment analysis of ML group and control.
