# Supplementary material for: Synergistic Antibacterial Mechanism of Mannosylerythritol Lipid-A and Lactic Acid on Listeria monocytogenes Based on Transcriptomic Analysis
Source: Foods. 2022 Sep 1;11(17):2660. doi: 10.3390/foods11172660 (PMC9455235; doi:10.3390/foods11172660)
Supplement: Supplementary file 1 [file foods-11-02660-s001.zip › Supplementary File/Supplementary Material.pdf]

Supplementary file

Figure S1 Bubble plot of GO enrichment analysis for ML group and control.

Figure S2 KEGG histogram enrichment analysis of ML group and control.

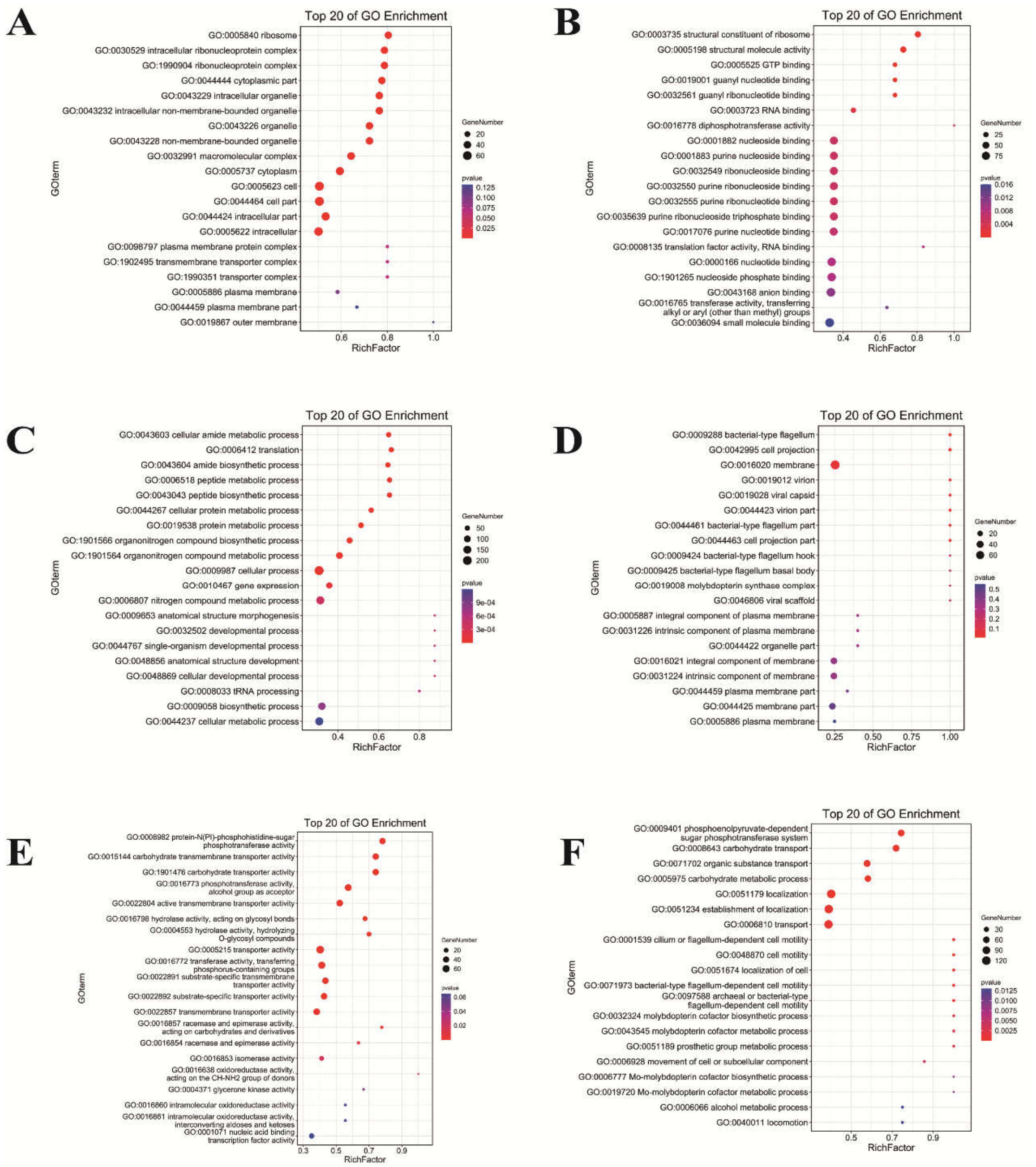

Figure S1. Bubble plot of GO enrichment analysis for ML group and control. Three main GO categories in up-regulated genes: (A) cellular component (CC), (B) molecular function (MF), (C) biological process (BP). Three main GO categories in down-regulated genes: (D) cellular component (CC), (E) molecular function (MF), (F) biological process (BP).

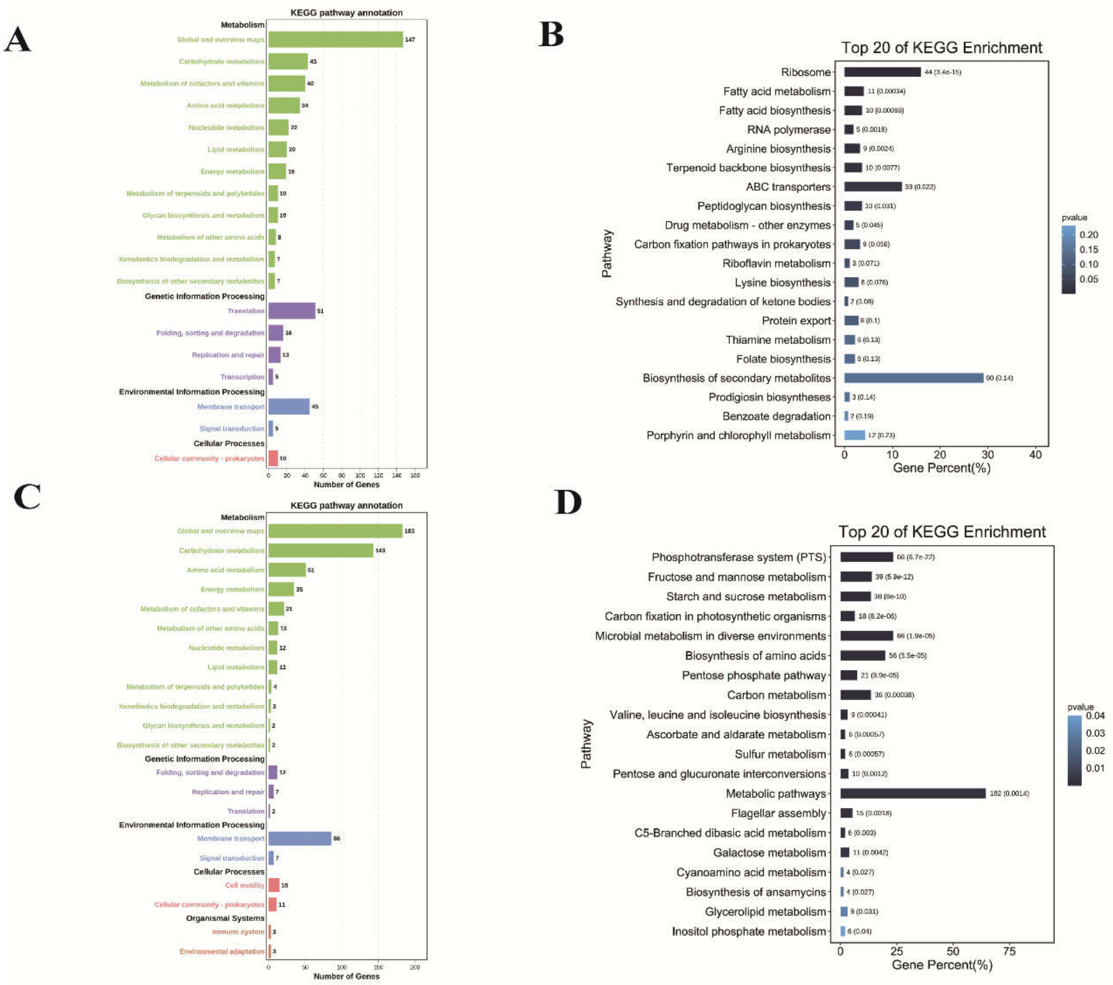

Figure S2. KEGG histogram enrichment analysis of ML group and control. Statistical plots of KEGG pathway B class classification in up-regulated (A) and down-regulated genes (C). Bar graphs of the top 20 significantly enriched pathways in up-regulated (B) and down-regulated genes (D).
